# A heart failure network model to improve outcome and trans‐sectoral guideline‐directed medical treatment utilization

**DOI:** 10.1002/ehf2.15434

**Published:** 2025-10-10

**Authors:** Christina Paitazoglou, Dominik Jurczyk, Matthias Mezger, Felicitas Lemmer, Bernhard Schwaab, Patricia Grube, Thomas Helms, Bettina Zippel‐Schultz, Buntaro Fujita, Roland Tilz, Thomas Stiermaier, Christian Frerker, Stephan Ensminger, Ingo Eitel

**Affiliations:** ^1^ University Heart Center Lübeck, Medical Clinic II (Cardiology, Angiology, Intensive Care Medicine) University Hospital Schleswig‐Holstein (UKSH) – Campus Lübeck Lübeck Germany; ^2^ German Center for Cardiovascular Research (DZHK), Partner Site Hamburg‐Kiel‐Lübeck Lübeck Germany; ^3^ Curschmann Klinik Timmendorfer Strand Germany; ^4^ German Foundation for the Chronically Ill Berlin Germany; ^5^ University Heart Center Lübeck, Clinic for Heart‐ and Thoracic Surgery University Hospital Schleswig‐Holstein (UKSH) – Campus Lübeck Lübeck Germany; ^6^ University Heart Center Lübeck, Clinic for Rhythmology University Hospital Schleswig‐Holstein (UKSH) – Campus Lübeck Lübeck Germany

**Keywords:** Chronic heart failure, Epidemiology, Guideline‐directed medical therapy, Heart failure network, Health service research

## Abstract

**Aims:**

Heart failure (HF) is a major cause of hospitalization, mortality and healthcare costs. Reducing its socioeconomic burden is a key global public health priority. HF networks are recommended to improve screening and management of HF patients. We developed and implemented a multi‐sectoral HF network in Northern Germany aimed at optimizing patient outcomes.

**Methods and results:**

A regional HF network was established by integrating 12 pre‐existing local networks into a state‐wide, multi‐sectoral HF network. Data from HF‐coded patients were analysed for two time periods: pre‐implementation (2018–2020) and post‐implementation (2021–2023). Patient trajectories through the healthcare system were examined using both inpatient and outpatient datasets. We report on the network's implementation across urban, island and rural areas, along with associated challenges and benefits. A roadmap of HF patient trajectories was created, identifying key healthcare entry points and informing a three‐pillar theory of change to address the national HF burden.

Post‐implementation, outpatient treatment cases increased markedly (2018–2020 *n* = 1237 vs. 2021–2023 *n* = 2563; +101.3%, *P* < 0.001), as did referrals from specialists (2018–2020 *n* = 290 vs. 2021–2023 *n* = 434, +49.7%, *P* = 0.013), general practitioners (2018–2020 *n* = 369 vs. 2021–2023 *n* = 435, +17.9%, *P* = 0.26), and inpatient admissions (2018–2020 *n* = 2342 vs. 2021–2023 *n* = 2608, +20.7%, *P* = 0.03). HF rehospitalization rates showed no significant difference yet despite a positive trend (2018–2020 20.3% vs. 2021–2023 17.9%; *P* = 0.295), while in‐hospital mortality remained stable (2018–2020 8.8% vs. 2021–2023 10.2%; *P* = 0.1).

**Conclusions:**

Implementation of a novel multi‐sectoral HF network enabled the analysis of patient trajectories and identification of areas for improvement in HF care. Observed shifts in referral patterns and increased treatment activity indicate early positive trends that support the potential of such networks in enhancing HF management and reducing disease burden.

## Graphical abstract

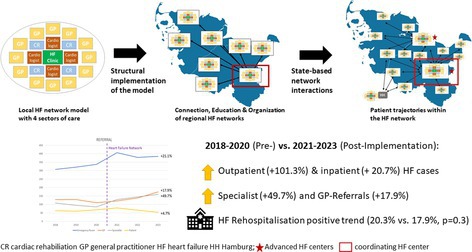

## Introduction

Heart failure (HF) is a highly prevalent clinical syndrome with increased mortality, morbidity and associated with poor quality of life.^1^, ^2^ Currently, the incidence of HF in Europe is 5/1000 person‐years, affecting 1–2% of adults or even >10% in those aged over 70 years or older, and therefore projected to increase in the future due to the ageing of the population.^1^ Additionally, data from the US inpatient sample demonstrate that the number of HF hospitalizations is more than doubled from 2008 to 2018 and continues to rise.^3^, ^4^ After initial diagnosis, HF patients are hospitalized once every year on average.^5^ Consequently, hospitalization rates are high and the burden on the health care system contributes to 70–80% of the cost of HF care in developed countries.^6^, ^7^ As inpatient costs are a major driver of the cost burden, HF networks that improve HF management and the high rehospitalization rates may help to lower healthcare costs.

The last three decades have seen a dramatic progress in the management of HF patients; therefore, an early diagnosis and implementation of guideline‐directed medical therapy (GDMT) is of utmost importance. Unfortunately, the diagnosis of HF is often missed or delayed in the general community, leading to delayed treatment and potentially avoidable (re)hospitalizations and deaths.^8^ Large real‐world registries like the Swedish Heart Failure Registry, the EVOLUTION‐HF registry or TITRATE‐HF demonstrate delayed HF diagnosis and GDMT initiation, as well as missed target doses and GDMT discontinuation over time that remain high.^9^, ^10^, ^11^, ^12^ International and German guidelines clearly recommend the implementation of multidisciplinary HF management programmes and cardiac rehabilitation (CR) programmes to ensure accurate diagnosis and appropriate GDMT with suitable follow‐up.^1^ To optimize HF care in Germany, the German Cardiac Society (DGK) defined the profile and certification of dedicated heart failure units.^13^ Despite the defined European and national guideline recommendations, HF management varies strongly depending on the local resources and national geographic structures, failing to implement the latest guideline recommendations.

We aimed to shape a HF network model potentially applicable in every country and to implement a state HF network in a wide region with urban, island and rural regions with governmental support. This allows a trans‐sectoral recognition of HF care to optimize future HF treatment.

## Methods

**Figure 1:**
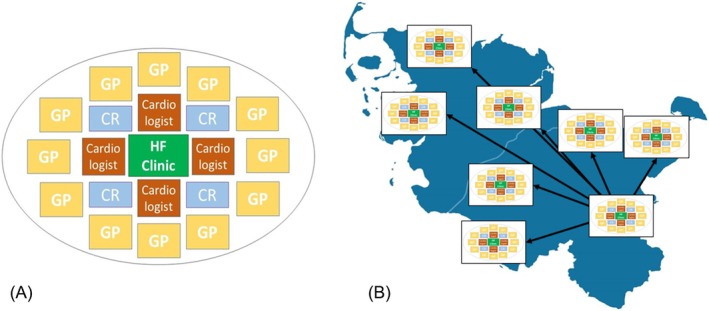
(A) Structure of the components of a local heart failure network model. (B) Implementation and structure of a state HF network with a central network coordination.

A model of a local HF network is depicted in *Figure* *1* *A*. Four different sectors of care are united to form a local structure and to treat HF patients by sharing common treatment structures in the form of standard operating procedures (SOPs) based on the European guideline recommendations: (i) clinic, (ii) general practitioner (GP), (iii) outpatient cardiologist and (iv) CR; offering comprehensive HF treatment. This single structure can be implemented in different regions or systems but needs to be adapted to the specific demands and capabilities of the pre‐existing environment integrated in a commonly used (but adjustable) SOP. In a second step, local networks were connected by a central network (nucleus) unifying them to a state or country network (*Figure* *1* *B*). With governmental support (Ministry of Justice and Health, Schleswig‐Holstein), we structured the network and defined aims (*Table* S1).

We analysed secondary patient data derived from in‐ and outpatient databases in the leading central network, an all‐comer HF patient population from January 2018 to December 2023. After establishment and implementation of these defined network structures, we assumed that patient data analysis in a network model will be representative for others and easily transferable after connection to the central network and structured training, a present, continuously ongoing implementation process in the State Department and northern part of Germany.

We extracted information about patient referral characteristics and treatment outcomes from the in‐ and outpatient databases. Comparisons were made in the general population 3 years before and after HF network establishment (2018–2020 vs. 2021–2023). Baseline characteristics, risk factors as well as discharge, rehospitalization and mortality rates were analysed in this vulnerable population. This organizational advanced HF structure aims to be a blueprint for the claimed national structure and spread across Germany.^14^ Statistical analysis was performed using SPSS version 29 (SPSS Software, IBM, Armonk, NY, USA). We analysed baseline and rehospitalization variables using the paired t‐test or Wilcoxon signed‐rank test, where appropriate. Paired comparisons were discriminated by the HF network establishment in 2021, dividing the observational time into equal parts of 3 years (2018–2020 vs. 2021–2023). A *P*‐value <0.05 was statistically significant, 95% confidence interval. Results are reported as median with standard deviation. HF rehospitalizations are reported as counts of first occurrence. Missing clinical variables were left out in the specific analysis.

## Results

### Implementation of the network

The DGK defined a heart failure unit certification initiative in Germany 2016.^13^ There are still boundaries for this certification (i.e., requirements for the certification process and a billing process for each certified unit), and we observed many local HF sectors still being uncertified. HF management and treatment showed significant differences between the analysed local HF networks depending on local resources and national geographic structures, mainly lacking a trans‐sectoral commonly used treatment structure integrating both the GP and CR sector.

After defining the role model of a local network structure, incorporating the four elementary sectors of care (GPs, outpatient cardiologists, clinics and CR), we organized further local networks in the state department and wider region of Northern Germany accordingly. A trans‐sectoral commonly used SOP describing GDMT implementation and continuation, adapted to the regional situation and local resources, was implemented in every local network as a working base. This SOP was jointly developed by leading cardiologists from the network, CR cardiologists and GPs within a dedicated working group, according to the current European guidelines and adjusted for the optimal implementation to the national cardiologic society guidelines (DGK) and the National Disease Management Guideline Program (NVL Program), which is a joint initiative of the German Medical Association (BÄK), the National Association of Statutory Health Insurance Physicians (KBV), and the Association of the Scientific Medical Societies in Germany (AWMF) aiming at promoting quality in healthcare. Regional networks were connected to form the HF state network (*Figure* *1* *B*), in total 12 networks spread over Northern Germany.

### Patient trajectories in the network

**Figure 2:**
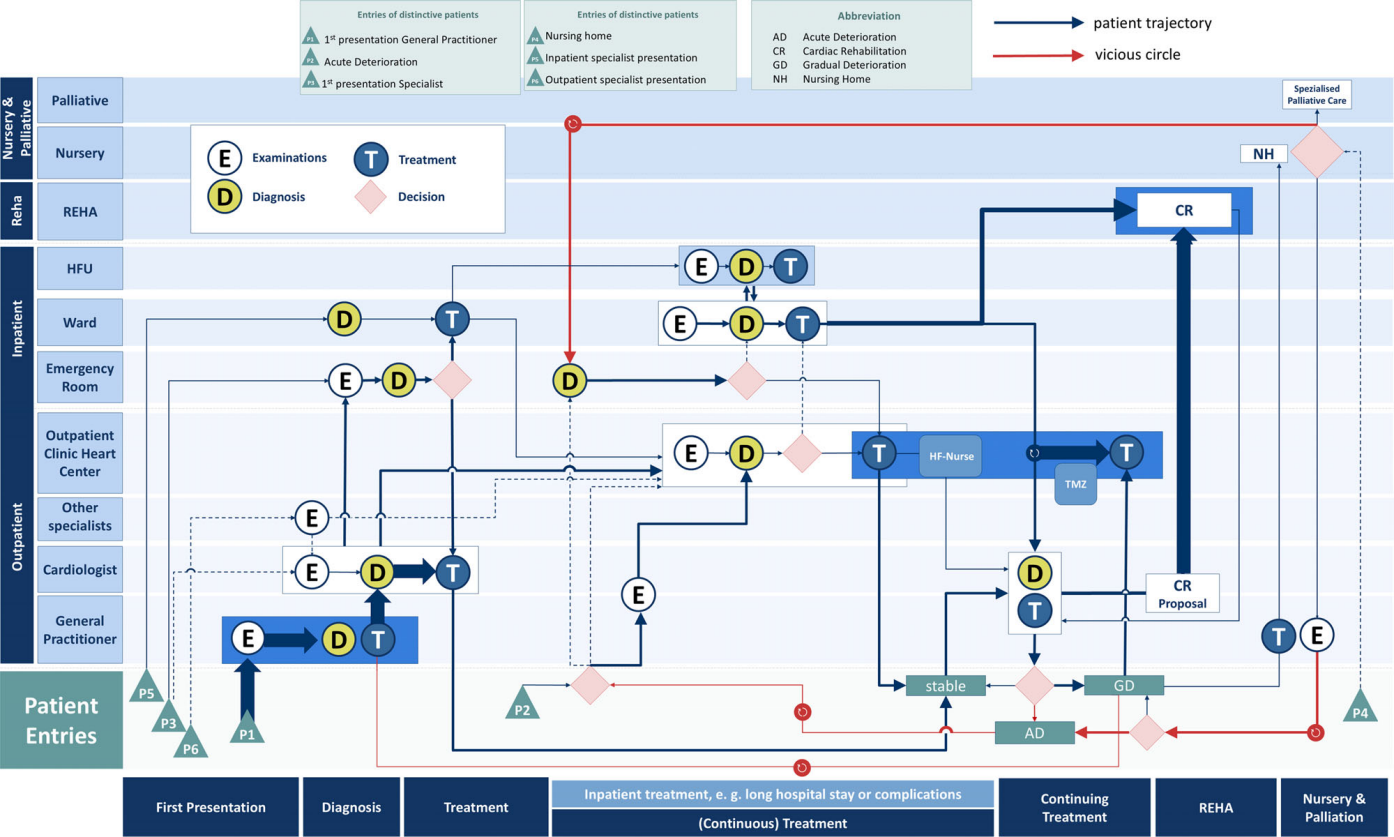
Heart failure network entry points and patient trajectories in the network after application of the 3‐pillar theory of change. This roadmap of patient trajectories was established to identify the entry points of HF patients into the healthcare system. On the *y* axis are all stakeholders in HF care, on the *x* axis the time in diagnosis, treatment and discharge care. Vicious circle of rehospitalization are depicted in red lines, three changes to improve therapy and prognosis in dark blue boxes.

In a second step, we analysed in‐ and outpatient databases with a focus on referral patterns and established a roadmap of HF patients identifying six entry points in the healthcare system (*Figure* *2*). Entry points into the HF network with HF diagnosis were (i) first presentation to the GP with symptoms of HF (P1), (ii) acute deterioration and referral to the emergency department (ER) without initial HF diagnosis in the community or GDMT (P2), (iii) first presentation with HF symptoms to an outpatient cardiologist, without GP referral (P3), (iv) patients from nursing homes with deterioration and direct hospitalization (P4) and (v–vi) in‐ or outpatient referral from specialists other than cardiologists (P5 and P6).

Weak points were numerous first self‐presentations in the ER (P2), delayed ambulatory HF diagnosis and treatment in P1, such as long specialist waiting times and direct referrals to the ER (P2) without early GDMT initiation.

### 3‐pillar theory of change

**Figure 3:**
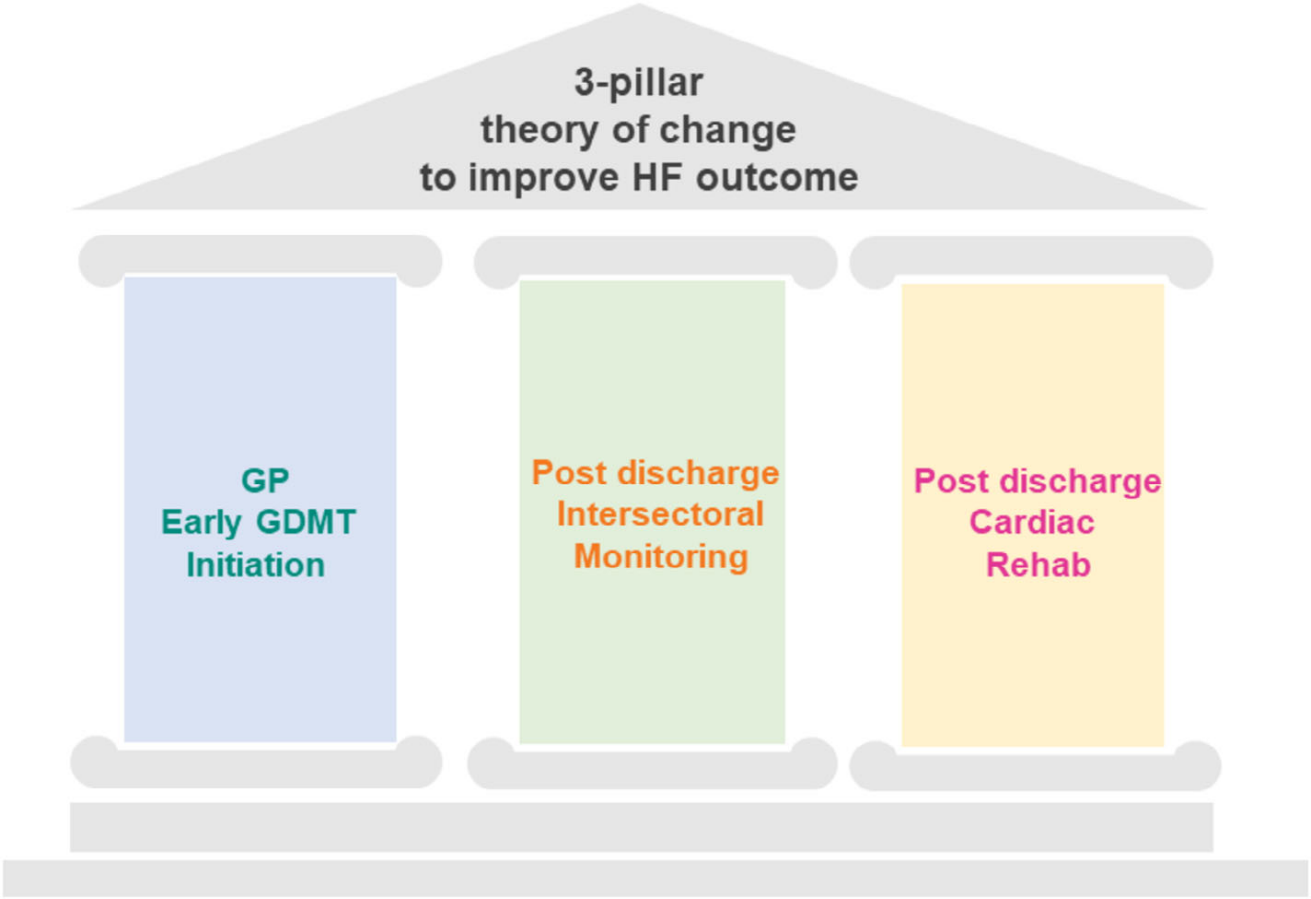
The 3 pillar‐theory of change.

A 3‐pillar theory of change was developed to tackle the HF burden, including multisectoral cooperation and strengthening specific entry points into the network (*Figure* *3*). First, hospitalization rates should be reduced by early diagnosis and GDMT initiation by GPs (P1) within the network and easy referral to an urgent HF care consultation. Secondly, intersectoral HF surveillance options, that is, HF nurses or telemonitoring, should be strengthened to avoid HF deterioration. Third, the awareness and utilization of HF rehab programmes should be empowered.

### Change in patient trajectories after network implementation

**Figure 4:**
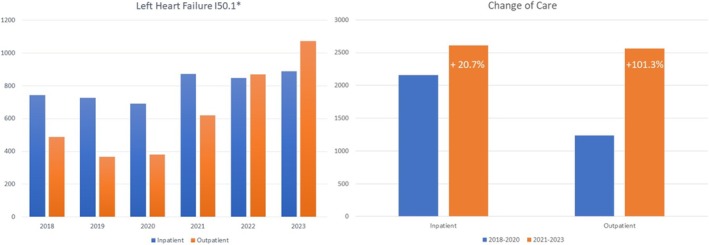
In‐ and outpatient left heart failure cases before and after network implementation.

**Figure 5:**
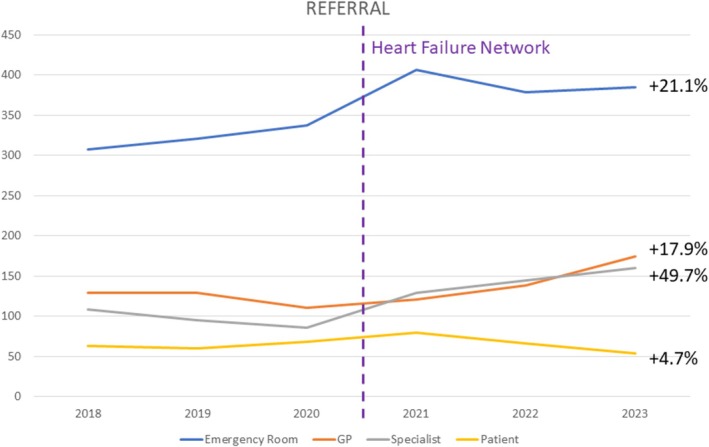
Change in referral patterns after network implementation.

The development of in‐ and outpatient HF treatment cases from 2018 to 2023, coded as left HF German version of International Classification of Diseases (ICD)‐10 I50.1*, are shown in *Figure* *4*. We observed increased in‐ and outpatient HF cases and treatments in the advanced HF centre after network implementation: inpatient *n* = 744 in 2018, *n* = 725 in 2019, *n* = 691 in 2020, *n* = 873 in 2021, *n* = 847 in 2022 and *n* = 888 in 2023 and outpatient *n* = 488 in 2018, *n* = 368 in 2019, *n* = 381 in 2020, *n* = 620 in 2021, *n* = 870 in 2022 and *n* = 1073 in 2023. In‐ and outpatient treatments rose by +20.7% (*P* = 0.03) and +101.3% (*P* < 0.001), respectively. Moreover, the pattern of referrals for inpatient HF cases changed (*Figure* *5*). Since the introduction of the regional HF network (2021–2023), first presentations via outpatient specialists increased by 49.7% (2018–2020 *n* = 290 vs. 2021–2023 *n* = 434, *P* = 0.013) and GPs by 17.9% (2018–2020 *n* = 369 vs. 2021–2023 *n* = 435, *P* = 0.26), but also in the emergency room by 21.1% (2018–2020 *n* = 966 vs. 2021–2023 *n* = 1170, *P* = 0.04). Self‐presentations were similar without a relevant change (+4.7%, 2018–2020 *n* = 191 vs. 2021–2023 *n* = 200, *P* = 0.72).

**Figure 6:**
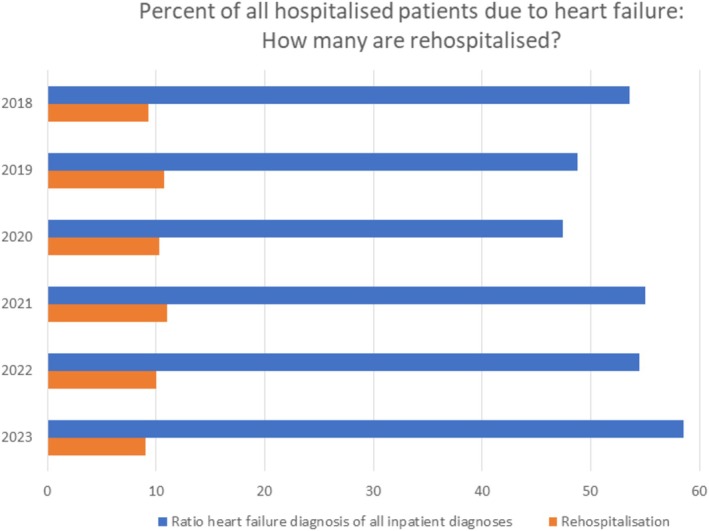
Percentage of (re‐)hospitalized heart failure patients.

HF hospitalization was the most common reason for hospitalization (>50% of all inpatient cases) over the past 6 years (*Figure* *6*). 29.6% of all HF patients experienced HF rehospitalization within 6 years. The HF rehospitalization rate per year was 20.3% before the HF network implementation and 17.9% after establishment (*P* = 0.295). In‐hospital mortality rates were comparable (2018–2020 vs. 2021–2023: 8.8 vs. 10.2%, *P* = 0.1) in this period.

## Discussion

Improving outcomes in HF is challenging despite remarkable advances in the field of medical and interventional HF treatment. Implementation of intersectoral HF networks is mandatory to warrant broad access to GDMT and to avoid suboptimal utilization of HF therapies in our daily practice. Clinical practice guidelines are crucial tools for healthcare professionals, offering management strategies based on thoroughly reviewed and current evidence. However, observational studies reveal the true gap in translation between the guidelines and implementation.^9^, ^10^, ^11^, ^12^ This discrepancy results in missed opportunities to lower morbidity, mortality and unplanned, inefficient healthcare use. Comprehensive intersectoral HF network models are the key to solving this issue.

We demonstrate the implementation, challenges and potential benefits of such a HF network in a wide region with urban, island and rural regions in Germany. In a second step, we established a trajectory map of HF patient entry points and vicious circles in the healthcare system to develop a 3‐pillar theory of change with strategies to tackle the national HF burden, including multisectoral cooperation and strengthening specific entry points into the network. Third, we analysed patient trajectories in the HF network model. Since the introduction of the HF network, an increase in in‐ and even more outpatient cases was observed. In addition, the outpatient HF clinic was facing a high demand for GP and specialist referrals. HF rehospitalization rates showed a positive trend without significant differences after establishment; in‐hospital mortality rates were comparable in this short period of time.

### The role network model

A widely accepted model of three different organizational levels—advanced HF centre, HF clinic, HF cardiologist—constituted as the infrastructural framework for the development of our HF model.^1^, ^2^, ^13^ The DGK defined the profile and certification procedure of HF units.^13^ Yet to date, many sectors remain uncertified; the CR and GP sectors were not included, and local structures vary. Therefore, it is important to build HF networks according to the recommendations of national and international societies to implement GDMT in a wide region according to common standards, preventing regional hierarchies and improving outcomes. Taking the national guidelines into consideration, we observed four main sectors incorporated into the structured HF treatment process: the GP, the outpatient cardiologist, the clinic and the rehabilitation sector. Forming these four sectors into a local HF network structure enables this model to expand to different regions or countries as these sectors are elementary points of HF care. National certification procedures should be supported within this model and supervised by a local HF network nucleus, that is, the nucleus of our described HF network model.

Unfortunately, the certification process to date is limited to general cardiologists and clinics despite GPs being an elementary HF treatment sector. Initial patient referral with de novo HF symptoms and elevated HF biomarkers, treatment initiation and defining the patient trajectory is often tackled mainly by this sector; many patients who present to the hospital or are diagnosed with HF for the first time have been previously referred to their GP with symptoms suggestive of HF yet did not receive appropriate treatment.^15^ By adopting the FIND‐HF mnemonic (Fatigue, Increased water accumulation, Natriuretic peptide level testing, Dyspnoea) in a clinical consensus document by the Heart Failure Association of the European Society of Cardiology (ESC), healthcare professionals can attain a higher level of suspicion for HF and have a lower threshold for making biomarker measurements to detect HF and start early treatment with a sodium‐glucose cotransporter‐2 inhibitor (SGLT2‐inhibitor), if there are no contraindications and diuretics in case of congestion.^15^ Furthermore, early post‐discharge management, short‐notice GDMT adjustment or referral with worsening HF (WHF) is also widely stemmed by this sector. Therefore, the incorporation of the GP sector in the HF network structure is warranted and was performed in our prescribed role model.

HF network SOPs and the structured discharge treatment process with standardized follow‐ups and up titration according to the current guidelines was commonly shared.^2^ Outpatient follow‐up requires consequent follow‐up; this stresses outpatient resources with limited specialist appointment capacity when tackled by one sector alone. Therefore, we standardized the follow‐ups according to local resources and implemented all sectors in the follow‐up process, including GPs and CR centres for the vulnerable posthospitalization phase. This was supported by follow‐up calls from heart failure nurses and the involvement of telemedicine centres within the leading model network. As a result, we observed no shortage of appointments in the outpatient cardiology sector for patients with other conditions requiring regular follow‐up. Unfortunately, reimbursement for HF nurses is not yet standard in Germany, and access to telemedicine centres remains limited and region dependent.

We included the CR clinics, another less defined sector so far, into our HF network role model, as the HF trajectory started with an acute HF hospitalization, a vulnerable phase for HF patients irrespective of the phenotype and left ventricular ejection fraction (EF) and needs intensive follow‐ups because of high re‐admission rates in the first month of discharge.^16^ Treatment of patients in an HF network may improve outcomes; therefore, the current ESC guidelines recommend a multidisciplinary HF network with CR programmes for patients with HF as a class I recommendation to improve outcomes and reduce the rates of hospitalizations.^1^, ^17^, ^18^

The 2021 ESC HF guideline recommendation for medical therapy is now (with the ESC HF guideline update 2023) further supported by the published STRONG‐HF trial with rapid up‐titration in HF patients leading to an improved outcome compared to standard of care.^11^ It is undoubted that the protocol as performed in STRONG‐HF, with weekly follow‐ups, is not feasible in a real‐world European and German setting and therefore, new strategies to improve GDMT remain urgently needed.^11^ Thus, we implemented our own follow‐up strategy in our shared SOP (2 weeks and 3 months after HF hospitalization); regular laboratory check‐ups are carried out by the GPs to avoid discontinuation of GDMT.

### Patient trajectories

By analysis of patient trajectories in our network, we observed weak points, that is, numerous first self‐presentations in the ER, delayed ambulatory HF diagnosis and treatment, such as long specialist waiting times and direct referrals to the ER without early GDMT initiation. CR programmes or post‐discharge trans‐sectoral patient telemonitoring were sparsely used. HF patients' surveillance in HF networks may be supported by telemonitoring. Several randomized studies such as LINK‐HF, TEMA‐HF1, TIM‐HF2, BEAT‐HF, CARDIO‐BBEAT, HERMeS or studies in patients with implanted devices (latest MONITOR‐HF) have shown a reduction in HF hospitalizations and mortality and have proven the clinical benefit of telemonitoring for HF patients.^19^, ^20^, ^21^, ^22^, ^23^, ^24^, ^25^, ^26^ Access to telemonitoring is still very limited; they are located in urban regions and the total number of telemonitoring centres is low compared to the high HF patient burden population. In our 3‐pillar theory of change, telemonitoring is typically coordinated by telemedicine centres in collaboration with cardiologists and HF nurses. Legal challenges include data protection (GDPR), medical liability and clearly defined responsibilities among providers. In Germany, telemonitoring for eligible HF patients is reimbursed through statutory health insurance, but funding for HF nurses remains inconsistent. Broader, standardized compensation models are still under discussion.

### 3‐pillar theory of change

A 3‐pillar theory of change was developed to tackle the HF burden, including multisectoral cooperation and strengthening specific entry points into the network (*Figure* *3*). First, hospitalization rates should be reduced by early diagnosis and GDMT initiation by GPs. Early initiation of GDMT was proven effective in preventing HF hospitalization events very shortly, even days after treatment started.^27^, ^28^, ^29^, ^30^ Furthermore, two different SGLT2‐I have been found to reduce the risk of HF hospitalization and cardiovascular death, irrespective of the left ventricular EF, revolutionizing the HF field and making GDMT initiation easy without the need of an echo.^2^ Steps across the whole HF trajectory and sectors, from early stages, through critical stages to stable stages and up to terminal stages need to be clearly defined in network‐accepted SOPs, multisectoral from primary to hospital care and therefore widely spread and used. To improve early diagnosis of HF and timely initiation of GDMT by GPs, a SOP (*Figure* S1A,B, adapted from^8^, ^15^) was developed and implemented during a team‐based network sector leader workshop. The SOP was based on the REVOLUTION‐HF trial and supports patient profiling according to symptoms and elevated biomarkers (NT‐proBNP levels) in the primary care setting.^31^ It includes stratified recommendations for specialist referral based on biomarker levels (e.g., NT‐proBNP >2000 ng/L warrants referral within 1 week). Early GDMT initiation focuses on starting SGLT2‐I and diuretics based on the patient's symptoms of congestion.

To strengthen the P1 entry point (GP referral), SOPs based on the current guidelines are shared and commonly used in the network by all sectors, and regular intersectoral meetings within the local network unit are held to emphasize the importance of GDMT. Secondly, intersectoral HF surveillance options, that is, HF nurses or telemonitoring, should be strengthened to avoid HF deterioration. Finally, the awareness and utilization of HF rehab programs should be empowered; a seamless transition to CR would be preferable to prevent early readmission. To strengthen this pillar, we implemented the CR sector in the local network model. With this 3‐pillar theory, the P2 entry point (ER referral) is expected to decrease, the number of (re)hospitalizations should be reduced, and the outcome improved.

Regular cross‐sector meetings across the network are essential to ensure the consistent use of SOPs. In our network model, the university hospital serves as the central coordinating body, responsible for the design and continuous updating of SOPs, as well as for maintaining a digital platform. The implementation of a digital platform offering access to all SOPs was financially supported by the Ministry of Health for the State Department of Schleswig‐Holstein. Most of the administrative workload occurs at the beginning and is then continuously updated in smaller steps, integrated into the routine operations of the university hospital, which is also responsible for coordinating the network at the state level.

### Prevention of worsening heart failure

Current estimations of HF individuals are globally around 60 million, in Europe 20 to 30 million, and in the United States 6.7 million.^17^, ^32^ The classification of WHF emphasizes the extremely high risk of HF patients compared to other cardiovascular diseases, such as coronary artery diseases, and claims attention in this vulnerable population.^16^ Within the state‐based HF network, SOPs are shared equally with all partners. The organizational structure works with HFA acronyms. For general screening, a new GP SOP was to introduced to FIND HF,^15^ and for the advanced HF population, I NEED HELP was used beside the four criteria of the ESC and HFA.^14^ The latter already demonstrated a high prognostic impact on the composite endpoint of all‐cause mortality or first HF hospitalization in the HELP‐HF Registry.^33^ The HF network implementation led to a sustainable effect in increasing numbers and steering the patient trajectories to the demand of outpatient colleagues, such as GPs. Their systematic screening in the general population is necessary and supports appropriate HF diagnosis and treatment over the whole natural course of HF.

We observed an increase in outpatient treatment cases with more referrals by specialists and GPs, along with an overall increase in elective patient referrals and advanced HF cases after network implementation. Another important contributing factor in improving patient trajectories during the network implementation was the publication of the ESC HF guidelines 2021 and the Update 2023.^1^, ^2^ Throughout the network, we constantly raised awareness of these guidelines to improve patient treatment and outcomes; the cross‐sector dissemination of this knowledge was appreciated by all participating sectors, making treatment more transparent. Raising awareness of the disease possibly contributed to the increased ER referrals we observed in the network. We cannot rule out that a contributing factor may be due to the ‘natural increase’ of patients with HF and that the observational study period was short.

Underutilization is common in the HF population: (i) GDMT in the eligible HF population,^10^, ^34^ (ii) cardiac resynchronization therapy (CRT) in persistent HF^35^ as well as (iii) heart transplantation and LVAD. As a consequence, patients do not have the projected mortality and HF hospitalization benefits.^34^ After 4 years without advanced HF treatment options like heart transplantation or LVAD, the mortality is up to 86%.^36^ Transferring this abstract model into reality, also in the developed countries, imposes lots of barriers, such as trans‐sectoral communication and care, structured follow‐ups, data availability and accessibility, and, if necessary, insurance companies. For example, Germany has to date no electronic health record, no automated data exchange between in‐ and outpatient care, and no national HF registry. Therefore, national demands vary, and there is a huge need to develop an advanced HF care network structure to be implemented across Germany. Hence, this analysis imposes first positive signs from a state‐based HF network that coordination and education of local networks lead to higher HF treatment cases, earlier referral for HF specialist evaluation, and less HF rehospitalization.

## Limitations

The present study was an observational study with a limited sample size and its typical limitations, like the risk of confounding bias. The HF network model and 3‐pillar theory of change were developed in Northern Germany. Therefore, they may not be directly transferrable to other regions or countries without adjustments in the SOP, taking local resources into consideration. Randomized studies on this topic are not available, and the use of real‐world data on an HF collective of patients in a wide state department with rural, urban regions as well as islands is the strength of the study. This supraregional HF network model is likely beneficial for patient trajectories and follow‐ups but has not yet shown a definitive improvement in health outcomes including MACE, mortality, symptom burden and severity of disease. General dynamics could have influenced the findings that are non‐related to the presence or absence of the HF network. A further limitation was the use of HF ICD codes for general conclusions on the treated population. They may vary from typical chronic HF patients to valve diseases or rhythmic disorders. Moreover, no differentiation among HF with reduced, mildly reduced and preserved ejection fraction was possible. Rising numbers of HF cases in the health service records are self‐explanatory in the ageing population and their high incidence of HF. Nevertheless, we need structures like the HF network to guide outpatient colleagues and steer patient trajectories for timely appropriate HF diagnosis and treatment. A further limitation is the analysis of secondary patient data, including rehospitalization rates derived from in‐ and outpatient databases in the leading central network; we therefore did not include patient data outside our network and cannot rule out readmissions in these regions. Mortality rates were only analysed from in‐hospital databases, as outpatient data for mortality were not available and possibly incomplete in this real‐world setting.

The effects observed may be affected by confounding factors. We sincerely believe that a combination of factors led to the positive effects we observed after network implementation: firstly, the publication of the European guidelines in 2021 and their update in 2023; secondly, the implementation of the network structure; and thirdly, the ongoing efforts across the network to raise awareness and apply the current guidelines in real‐world practice, lastly with the help of a free accessible digital platform provided by the HF network (HeartConnect App).

## Conclusions

We describe a model of a trans‐sectoral HF network, possibly applicable in other countries and incorporating 4 elementary sectors of primary care in HF treatment. The analysis of patient trajectories with a 3‐pillar theory of change was developed to tackle the HF burden and is an important step to perform improvements in HF care. Further scientific evaluation is needed. In future, evaluation of changes should be assessed in the whole area in cooperation with the federal statistical office of Germany. The changes in health services and referral patterns show first positive signs by the state‐based HF network. Outcome data, such as HF hospitalization and mortality, show a positive trend as well but remain uncertain in the small cohort. The following years must prove continuation.

## Conflict of interest

No conflicts of interest to report.

## Funding

For this study, we received funding support from the Ministry of Justice and Health, Schleswig‐Holstein, Germany.

## Supporting information


**Figure S1.** General Practitioner Standard operating procedure (Page 1&2).
**Table S1.** Aims of a state HF network.
