# Supplementary material for: A heart failure network model to improve outcome and trans‐sectoral guideline‐directed medical treatment utilization
Source: ESC Heart Fail. 2025 Oct 10;12(6):4305–15. doi: 10.1002/ehf2.15434 (PMC12719809; doi:10.1002/ehf2.15434)
Supplement: Supplementary file 1 — Figure S1. General Practitioner Standard operating procedure (Page 1&2). Table S1. Aims of a state HF network. [file EHF2-12-4305-s001.docx]

**SUPPLEMENT**

**Figure 1.** General Practitioner Standard operating procedure (Page 1&2)


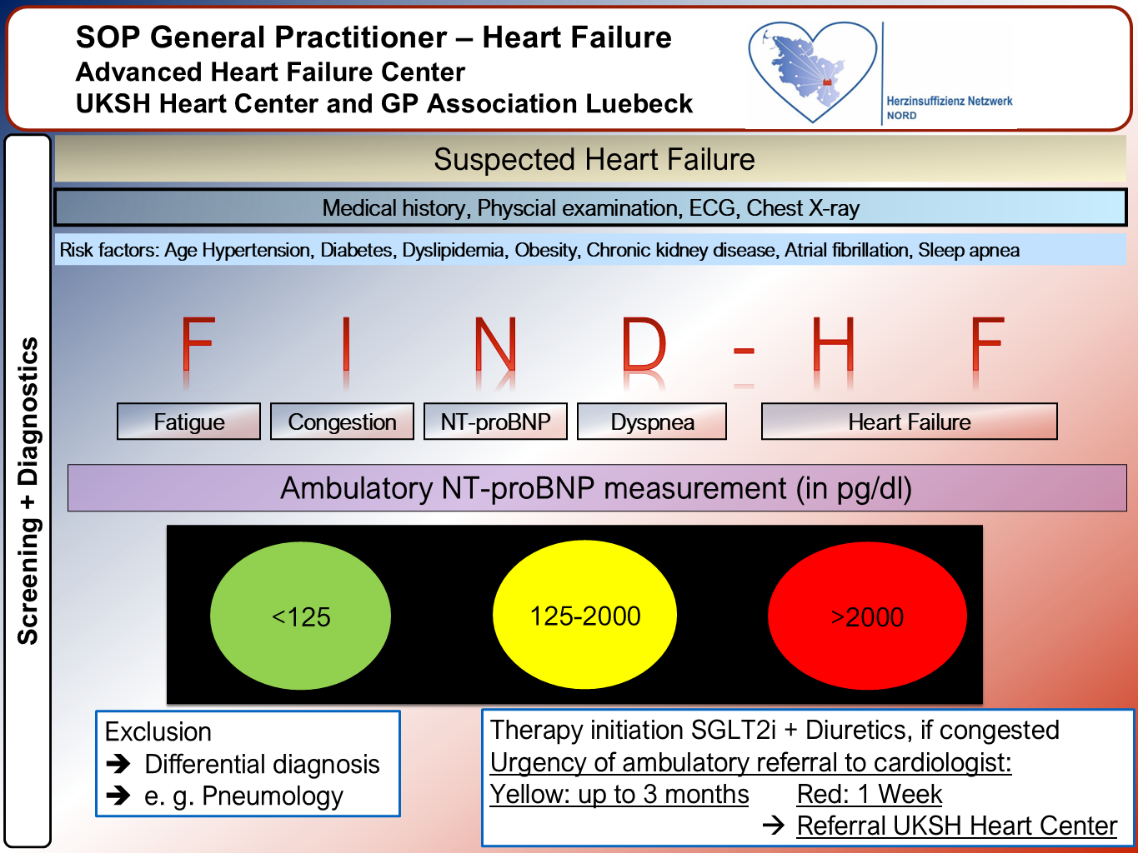

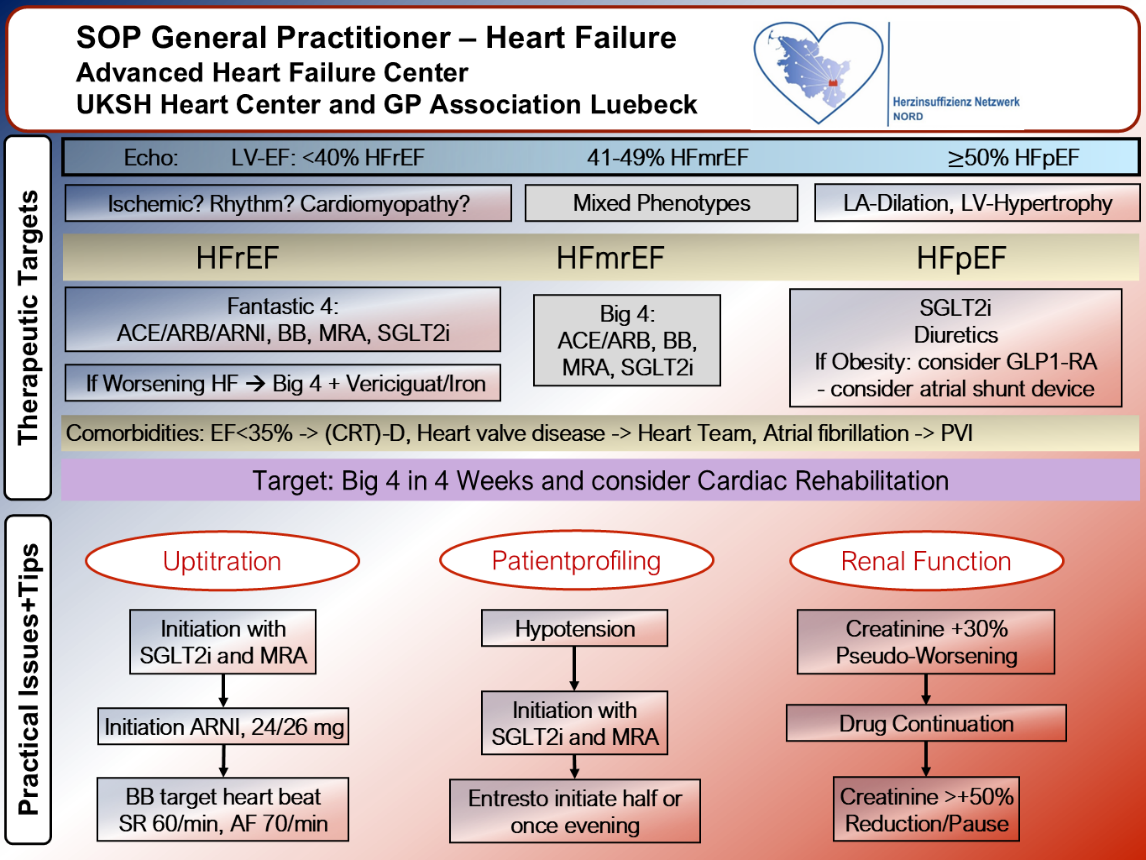


**Table 1.** Aims of a state HF network

| **Aim** |  |
| --- | --- |
| Intersectoral communication in a regional HF network | - The local network should have a coordinating nucleus defined by one advanced HF centre - HF centres should have a dedicated multidisciplinary team to manage patients with HF and define the sectors involved in the local network - Frequent intersectoral meetings for intersectoral and interdisciplinary communication - Established treatment patient flow regime for all stages from stable, to acute and advanced HF patients - Utilization of commonly used standard operating-procedures, which should be regularly updated according to the current guideline recommendations |
| Outpatient management of urgent care | - HF centres should define outpatient consultation hours for urgent HF cases with defined treatment slots, reserved for HF patients to avoid acute ER presentation - HF centres should raise the awareness of urgent HF care in the local network and might introduce a hotline for easy access |
| Define different follow-up options for patients with difficult access to outpatient clinics i.e. islands, rural regions | - Implementation of online video-follow-ups - Implementation of telephone-follow-ups - A network should include access to at least one telemonitoring center |
| Define in-hospital pre- and post-discharge management | - Structured in-hospital pre-discharge management with patient empowerment, heart failure-nurse led patient education - Hospitals should define post-discharge follow-ups i.e. after 2 weeks and 3 months by a cardiologist - Regular follow-ups up to six weeks should be assessed individually in patients at high risk and advanced heart failure |
| Intersectoral patient surveillance to prevent HF hospitalization | At least one or more of the following options should be available:   - Telemonitoring centres - Telephone contact to heart failure nurses - Invasive hemodynamic monitoring, i.e. CardioMEMS^R^ |
| Access to information about the local network | - The local network should implement a homepage or application with relevant information, SOPs |
